# Notch controls effector CD8^+^ T cell differentiation

**DOI:** 10.18632/oncotarget.4886

**Published:** 2015-07-17

**Authors:** Frédéric Duval, Mélissa Mathieu, Nathalie Labrecque

**Affiliations:** Maisonneuve-Rosemont Hospital Research Centre, Department of Medicine and department of Microbiology, Infectiology and Immunology, University of Montréal, Montréal, Québec, Canada

**Keywords:** Immunology and Microbiology Section, Immune response, Immunity, notch, infection, CD8 T cells, vaccination

CD8+ T cell recognition of foreign antigen induces the expansion of the few T cells bearing an appropriate T cell receptor and the acquisition of effector functions. These effector CD8+ T (Te) cells eliminate the pathogen by secretion of cytokines or by direct cytotoxicity. Following this initial clonal expansion, 90–95% of the Te cells die while the few others further differentiate into memory T (Tm) cells conferring long-term protection against future invasion by the same pathogen. Therefore, the success of a primary immune response requires proper control of cell fate to allow for the generation of functional short-lived effector cells (SLECs; CD127^lo^KLRG1^hi^) and memory precursor effector cells (MPECs; CD127^hi^KLRG1^lo^) that will differentiate into long-lived Tm cells. This differentiation choice is instructed by key transcription factors promoting either SLEC or Tm cell generation. High expression of the transcription factors T-bet and Blimp-1 governs SLEC differentiation while Eomes and Bcl-6 promote memory generation [1]. Although, we know that inflammatory cytokines, such as IL-12 and IL-2, regulates SLEC generation by inducing T-bet and Blimp-1 expression [1], other signals provided by the environment may also influence the SLEC/MPEC differentiation choice. Previous evidence suggested that the Notch signalling pathway, known to play a critical role in cell fate decision throughout development, has the potential to instruct CD8+ T cell fate during an immune response. This evolutionary conserved pathway is composed of 4 receptors (Notch1–4) and five ligands (Jagged1–2, Delta-like-1, -2, -3). The interaction of the receptor with its ligands leads to its proteolytic cleavage releasing the Notch intra-cellular domain (NICD) that will translocate to the nucleus where it associates with RBPJ. This allows for the assembly of a transcriptional activator complex that will induce the transcription of target genes to promote the proper differentiation choice. Furthermore, the expression of Notch1 and Notch2 by activated CD8+ T cells and the reported ability of the Notch signaling pathway to affect the transcription of key molecules controlling effector differentiation (T-bet and Eomes) and function (cytokines and cytolytic molecules) [2, 3] prompted us and the group of Amsen to decipher the role of Notch during *in vivo* CD8+ T cell response to acute infection and vaccination [4, 5].

Using mice where Notch1 and Notch2 are deleted only in mature CD8+ T cells, we have recently demonstrated that Notch signalling is intrinsically required for SLEC differentiation following *Listeria monocytogenes* (Lm) infection and dendritic cell (DC) vaccination [4]. Despite a decrease in SLECs, MPEC and memory T cell generation occurs normally. Decreased SLEC generation correlated with reduced CD25 (IL-2Rα chain) expression, for which sustained expression promotes SLEC differentiation [6]. However, in our models, defective SLEC differentiation occurs despite normal expression of the master transcription factors, T-bet and Blimp-1, known to be essential for SLEC generation [4]. The group of Amsen, using the Influenza viral infection model, also demonstrated that Notch signaling is essential for sustaining CD25 expression and SLEC differentiation [5]. Unlike us, T-bet and Blimp-1 expression was reduced in Notch-deficient effectors generated following Influenza infection [5]. Altogether, these studies have uncovered an essential role for the Notch signalling pathway in orchestrating the differentiation choice that CD8 T cells face following activation. The mechanism by which Notch signaling controls SLEC differentiation is still not fully understood. However, these two studies allow us to propose the following model where direct binding of the NICD to the *cd25* promoter is essential to sustain high expression of CD25, which is necessary for SLEC differentiation. In some immunisation models, Notch-deficient effectors express normal level of T-bet and Blimp-1 suggesting that the NICD collaborates with these transcription factors to optimally transcribes target genes regulating SLEC differentiation. This is in accordance with the role of the Notch signaling pathway during helper T cell differentiation where the NICD collaborates with the master transcription factors governing Th1, Th2 or Th17 differentiation [7].

Interestingly, these studies have also revealed a context dependent role for the Notch signalling pathway in the acquisition of effector functions. Indeed, following an Influenza infection, less CD8 T cells produced IFN-γ and those cells produced lower amount of granzyme B and perforin, which correlates with a higher viral load in the lung 10 days post-infection [5]. However, Notch-deficient effectors generated following Lm infection were fully functional as they express normal amount of IFN-γ and efficiently control a challenge with a lethal dose of Lm [4]. On the other hand, the lack of Notch signalling have a much drastic effects following DC vaccination, very few antigen-specific T cells produce IFN-γ, IL-2 and TNF-α and as a consequence were unable to control a challenge with a lethal dose of Lm [4]. This context dependent role for Notch signaling in CD8+ T cell response could occur due to difference in the inflammation level or duration of antigen presentation. We can speculate that Notch will contribute to the transcription of genes coding for effector molecules only when the infectious agent or vaccination regimen does not optimally induce, in antigen-specific CD8+ T cells, the expression of other transcription factors controlling effector functions.

Gaining insight into the regulation of the CD8+ T cell response by the Notch signaling pathway may provide new avenues to ameliorate the generation of potent CD8+ Te cells endowed with optimal cytokine production and thus beneficial for cellular therapies against cancer and infectious agents. On the other hand, inhibiting the Notch signaling pathway could favor memory generation, the cardinal feature of vaccination.
